# Exploring the common ferroptosis-related genes and molecular mechanisms in periodontitis and systemic sclerosis via integrated bioinformatics and experimental analysis

**DOI:** 10.3389/fcell.2026.1803091

**Published:** 2026-04-02

**Authors:** Shengchao Zhang, Shengwei Yang, Cui Ge, Chao Ji, Ruohan Lin, Wenjuan Xue, Dongxiu Liu, Fulin Chen

**Affiliations:** 1 Northwest University First Hospital, Northwest University, Xi’an, China; 2 Laboratory of Tissue Engineering, College of Life Sciences, Northwest University, Xi’an, China; 3 Provincial Key Laboratory of Biotechnology of Shaanxi, Northwest University, Xi’an, China; 4 Key Laboratory of Resource Biology and Biotechnology in Western China, Ministry of Education, School of Medicine, Northwest University, Xi’an, China

**Keywords:** bioinformatics, ferroptosis, machine learning, periodontitis, systemic sclerosis

## Abstract

**Background:**

Accumulating evidence suggests reciprocal risk factors between periodontitis (PD) and systemic sclerosis (SSc). Ferroptosis, an iron-dependent and immune-related form of cell death, has been implicated in both diseases, yet its shared molecular mechanisms remain largely unclear.

**Methods:**

Bidirectional Mendelian randomization (MR) analysis was first conducted to evaluate the potential causal relationship between PD and SSc. Gene expression datasets for PD and SSc were retrieved from the GEO database, and ferroptosis-related genes were obtained from FerrDb. Differential expression and WGCNA identified common ferroptosis-related differentially expressed genes (Co-FRDEGs) and common ferroptosis-related module genes (Co-FRMGs). Functional enrichment analyses were subsequently performed. The intersection of Co-FRDEGs and Co-FRMGs yielded candidate genes, which were further screened by three machine learning algorithms (LASSO, SVM-RFE, and Random Forest) to identify shared hub genes. Immune infiltration, single-cell RNA sequencing, regulatory network analysis (TF-miRNA), and drug prediction with molecular docking were further performed. In addition, preliminary *in vitro* experiments were conducted to validate the expression and potential ferroptosis-associated roles of the identified hub genes.

**Results:**

MR revealed an asymmetric causal association between PD and SSc. A total of 28 Co-FRDEGs and 63 Co-FRMGs were identified, and their intersection yielded nine candidate genes. Machine learning analysis predicted FNDC3B and NNMT as shared hub genes, exhibiting good diagnostic performance (AUC >0.75) in both discovery and validation cohorts. Immune infiltration analysis revealed multifaceted immune dysregulation in both diseases, while single-cell analysis confirmed cell type-specific expression of the two hub genes. Regulatory network analysis predicted GTF2E2 and three miRNAs as potential co-regulators. Drug prediction and molecular docking suggested thapsigargin as a potential lead compound. Furthermore, *in vitro* experiments demonstrated that FNDC3B and NNMT were significantly upregulated in PD- and SSc-like cellular models, and their silencing alleviated ferroptosis-associated cellular injury.

**Conclusion:**

This study highlights potential shared ferroptosis-related genes, regulatory networks, and candidate therapeutic compounds associated with PD and SSc, providing new insights into their molecular connections. However, as these findings are largely derived from bioinformatics analyses with preliminary experimental validation, further mechanistic and clinical studies are required to confirm them.

## 1 Introduction

Periodontitis (PD), a microbe-driven chronic inflammatory disease ranking sixth in global disease prevalence, is characterized by progressive destruction of periodontal supporting tissues, including the gingiva, periodontal ligament, alveolar bone, and cementum (Papapanou et al., 2018; Frencken et al., 2017). The etiology of PD involves a complex interplay between dysbiotic subgingival microbial communities and a dysregulated host immune response, resulting in chronic inflammation and subsequent tissue destruction (Abusleme et al., 2000). PD has surpassed its traditional classification as a localized oral disease and is now increasingly recognized as a modifiable risk factor for systemic disease. Numerous epidemiological and mechanistic studies have demonstrated a tight link between PD and the onset and development of various systemic diseases, including diabetes, respiratory disorders, cardiovascular diseases, neurodegenerative disorders, and certain malignancies (Isola et al., 2023; Hajishe and ngallis, 2015). These associations are biologically plausible due to shared genetic susceptibilities, environmental exposures, and common inflammatory and immune dysregulation pathways.

Systemic sclerosis (SSc), also known as systemic scleroderma, is a rare heterogeneous autoimmune rheumatic disease characterized by immune dysregulation, widespread vasculopathy, and progressive fibrosis of the skin and internal organs (Denton and Khanna, 2017). The clinical manifestations of SSc vary widely, ranging from limited cutaneous involvement to severe multi-organ complications, particularly affecting the lungs, gastrointestinal tract, heart, and kidneys (Geyer and Müller-Ladner, 2011). Its pathogenesis is complex and multifactorial, involving dynamic interactions among genetic susceptibility, environmental factors, endothelial dysfunction, aberrant immune activation, and fibroblast-driven extracellular matrix deposition (Denton and Khanna, 2017; Geyer and Müller-Ladner, 2011). Despite its low prevalence, SSc carries the highest mortality rate among autoimmune rheumatic diseases, with all-cause mortality risk tripling that of the general population (Elh et al., 2012). SSc remains a great life-threatening condition posing substantial clinical challenges.

Emerging evidence suggests a bidirectional and potentially causal association between PD and SSc (Ciurea et al., 2023). Patients with SSc have been reported to exhibit a higher prevalence and severity of periodontal disease compared to healthy individuals (Jung et al., 2017). Conversely, chronic periodontal inflammation may trigger systemic immune activation and extracellular matrix remodeling, potentially exacerbating SSc progression (Elimelech et al., 2015). Several shared pathogenic features have been identified in both conditions, including elevated levels of pro-inflammatory cytokines and chemokines such as TNF-α, IFN-γ, TGF-β1, IL-1, IL-6, and IL-17, which drive immune dysregulation and tissue destruction (Elimelech et al., 2015; Matarese et al., 2012). Additionally, receptor activator of NF-κB ligand (RANKL), a key osteoclastogenesis mediator in PD, is also upregulated in SSc, suggesting overlapping osteoimmunological pathways (Belibasakis and Bostanci, 2012; Gamal et al., 2018). Moreover, the fibrotic and vascular alterations characteristic of SSc may disrupt periodontal homeostasis and contribute to PD development (Yuen et al., 2014). Despite recent advances in understanding the molecular pathways underlying these conditions, the interactions between PD and SSc remain complex and require further investigation.

Ferroptosis, first described by Dixon et al. (2012), is a recently identified form of regulated cell death (RCD) that is mechanistically distinct from apoptosis, necrosis, autophagy, and other cell death types (Dixon et al., 2012). This process features iron-dependent accumulation of lipid peroxides and reactive oxygen species (ROS), driven by dysregulated iron metabolism, glutathione peroxidase 4 (GPX4) inactivation, and compromised antioxidant defenses (Dixon et al., 2012). Growing evidence suggests that ferroptosis contributes to various pathologies including cancer, neurodegeneration, renal disease, cardiovascular diseases, and autoimmune diseases (Jiang et al., 2021). In PD, oxidative stress and disrupted iron metabolism promote alveolar bone loss through ferroptosis-related mechanisms. For instance, ferroptosis promoted osteoclastogenesis by upregulating pro-inflammatory cytokines, accelerating periodontal degradation (Tang et al., 2024). Additionally, *porphyromonas gingivalis* can induce NCOA4-mediated ferritinophagy, aggravate ROS production, and trigger inflammatory responses, which collectively drive ferroptosis in periodontal ligament fibroblasts (PDLFs) (Guo et al., 2021). Similarly, in SSc, ferroptosis appears to participate in immune dysregulation and progressive fibrosis. ACSL4 upregulation facilitates M1 macrophage ferroptosis, amplifying inflammation and fibrotic processes in SSc (Cao et al., 2023). Moreover, recent research indicated that GPX4 suppression sensitizes SSc fibroblasts to ferroptosis, highlighting its fibrogenic role (Zhang F. et al., 2024). Taken together, these findings suggest that ferroptosis may serve as a shared pathological mechanism underlying the comorbidity between PD and SSc.

With advances in high-throughput transcriptomic and genetic analyses, identifying common transcriptional features between PD and SSc may provide valuable insights into shared pathogenetic characteristics of these two diseases. In this study, we first applied bidirectional Mendelian randomization (MR) analysis to evaluate the potential causal relationship between PD and SSc. Subsequently, we performed integrated bioinformatics combined with machine learning algorithm to identify ferroptosis-related shared biomarkers and pathways in PD and SSc from GEO database. Additionally, immune cell infiltration analysis and single-cell RNA sequencing (scRNA-seq) were conducted to characterize immune alterations and to delineate the cell type-specific expression patterns of key biomarkers. Moreover, regulatory networks involving transcription factors (TFs) and miRNAs were constructed, and potential therapeutic compounds targeting shared biomarkers were predicted. Finally, *in vitro* experiments were performed to validate the expression and functional roles of the identified candidate genes in disease-relevant cellular models. Overall, these findings may offer hints for exploring the biological mechanisms of ferroptosis and developing targeted therapies for PD and SSc comorbidity.

## 2 Methods

### 2.1 Datasets collection and ferroptosis-related gene acquisition

**FIGURE 1:**
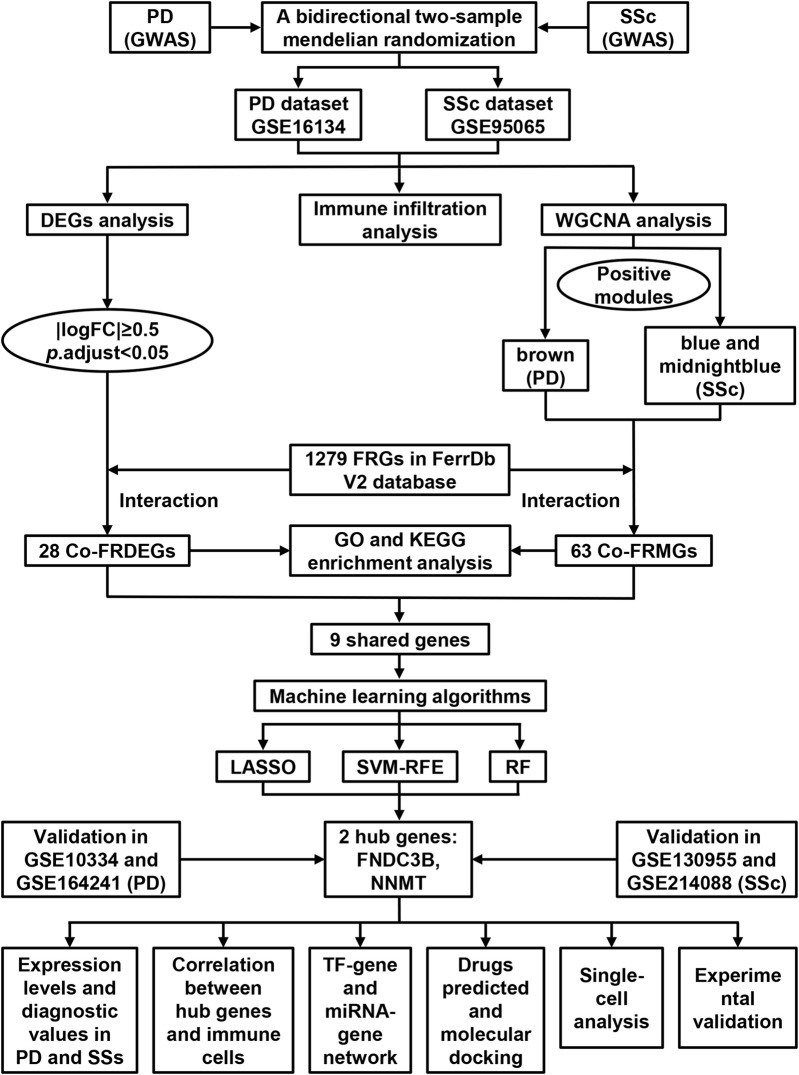
Flowchart depicting the analysis process of this study.

Figure 1 illustrates the overall study workflow. GWAS summary statistics for both exposure and outcome were obtained from the GWAS Catalog (https://www.ebi.ac.uk). Genetic instruments for PD were obtained from the GWAS dataset GCST008442, which included 975 individuals of European ancestry from the United States (394 periodontally healthy controls, 389 mild-to-moderate cases, and 192 severe cases). Summary statistics for SSc were derived from GCST90436638, comprising 175 British ancestry cases and 399,404 ancestry-matched controls recruited in the United Kingdom. Gene expression array datasets related to PD and SSc were downloaded from the Gene Expression Omnibus (GEO, https://www.ncbi.nlm.nih.gov/geo/). Four bulk transcriptomic datasets were included in this study: GSE16134 and GSE10334 for PD, GSE95065 and GSE130955 for SSc. Among them, GSE16134 (PD) and GSE95065 (SSc) were designated as discovery cohorts, while GSE10334 and GSE130955 served as validation cohorts for PD and SSc, respectively. In addition, two single-cell RNA sequencing datasets (GSE164241 for PD and GSE214088 for SSc) were analyzed to characterize cell-type-specific expression patterns. Detailed characteristics of each dataset are summarized in Table 1. In parallel, ferroptosis-related genes (FRGs) were obtained from FerrDb V2 database, which contained four categories: marker, driver, suppressor, and unclassified (Zhou et al., 2023). Merged categories yielded 1279 FRGs for subsequent analyses (Supplementary Table S1).

**TABLE 1:** Basic information of GEO datasets used in the study.

GSE number	Platform	Samples	Source type	Disease	Group
GSE16134	GPL570	241 cases and 69 controls	Gingival	PD	Discovery
GSE10334	GPL570	183 cases and 64 controls	Gingival	PD	Validation
GSE164241	GPL18573	8 cases and 13 controls	Gingival	PD	Single-cell analysis
GSE95065	GPL23080	18 cases and 11 controls	Skin	SSc	Discovery
GSE130955	GPL16791	48 cases and 33 controls	Skin	SSc	Validation
GSE214088	GPL16791	5 cases and 7 controls	Skin	SSc	Single-cell analysis

### 2.2 Mendelian randomization analysis

Single-nucleotide polymorphisms (SNPs) significantly associated with phenotype were selected as instrumental variables based on predefined association thresholds (*P* < 1 × 10^−5^ for PD and *P* < 5 × 10^−5^ for SSc) and pruned for linkage disequilibrium (r^2^ < 0.001 within a 10,000 kb window). Weak instruments were excluded based on F-statistic criterion (F < 10). Genetic variants were harmonized to align effect alleles between the exposure and outcome datasets. The inverse variance weighted (IVW) method was used as the primary estimator of causal effect, while MR-Egger regression and the weighted median approach were performed as sensitivity analyses. Heterogeneity among instrumental variables and potential horizontal pleiotropy were assessed using Cochran’s Q test and MR-Egger intercept test, respectively. MR estimates are reported as an odds ratio (OR). All MR analyses were conducted using the “TwoSampleMR” package.

### 2.3 Identification of differential expression genes (DEGs)

Raw datasets were processed using R software (version 4.2.3). Affymetrix microarray (GSE16134, GSE10334, and GSE95065) underwent RMA normalization using the “affy” package, while RNA-seq data (GSE130955) were processed with the “limma” package. Differential expression analysis for all datasets was conducted using the “limma” package with the cutoff threshold at |log2 fold change (FC)| ≥ 0.5 and adj. *P*-value <0.05. Volcano plots and heatmaps of DEGs were visualized using the “ggplot2” R package. Overlapping genes were identified via Venn diagrams.

### 2.4 Functional and pathway enrichment analysis

Gene Ontology (GO) analysis and Kyoto Encyclopedia of Genes and Genomes (KEGG) pathway enrichment analysis were performed using “clusterprofiler” package to elucidate the potential biological functions and signaling pathways associated with overlapping genes. GO analysis categorized gene functions into three domains: biological processes (BP), molecular functions (MF), and cellular components (CC), providing a structured vocabulary for gene annotation. KEGG pathway analysis is a comprehensive database resource used to identify enriched signaling and metabolic pathways associated with specific gene sets. Enriched results with adj. *P*-value <0.05 were considered statistically significant. Results were visualized using the “ggplot2” package.

### 2.5 Weighted gene co-expression network analysis (WGCNA)

WGCNA was conducted using the “WGCNA” package to identify biologically significant co-expression gene modules and explore gene network-phenotype associations. All expressed genes were included for network construction. A suitable soft-thresholding power (β) was selected for each dataset based on the scale-free topology criterion (R^2^ > 0.85), with β = 7 for PD and β = 12 for SSc, to ensure a biologically meaningful network structure. An adjacency matrix was computed and subsequently transformed into a topological overlap matrix (TOM) and the corresponding dissimilarity (1-TOM), which measures the network interconnectedness. Hierarchical clustering with dynamic tree cutting (minimum module size = 30, cut height = 0.25) identified module eigengenes (MEs). Pearson’s correlation test was used to calculate the correlation between ME and disease phenotypes. Additionally, gene significance (GS) and module membership (MM) linked modules to clinical features. Key modules showing strongest correlations were selected for downstream analysis.

### 2.6 Machine learning algorithms

To improve biological relevance and reduce potential false positives and feature dimensionality prior to modeling, overlapping genes between DEGs and disease-associated WGCNA modules were selected as candidate features. Next, three machine learning algorithms were applied: least absolute shrinkage and selection operator (LASSO), support vector machine-recursive feature elimination (SVM-RFE), and random forest (RF). LASSO regression was conducted using the “glmnet” package by constructing a logistic regression model, where 10-fold cross-validation was used to determine the optimal regularization parameter (λ), and lambda. min was selected as the optimal value (Tibshirani, 2011). SVM-RFE was implemented via the “e1071” package, which recursively eliminated the least informative features to identify the optimal subset of genes based on classification performance (Huang et al., 2014). RF were performed using the “randomForest” package, ranked genes according to their importance in predicting disease status by constructing multiple decision trees (Blanchet et al., 2020). Specifically, genes with a relative importance exceeding a threshold of one are considered key feature genes. Finally, the intersecting genes identified by all three algorithms were defined as hub genes and selected for subsequent analysis.

### 2.7 Expression analysis and diagnostic effectiveness evaluation of shared hub genes

The expression levels of hub genes between disease and control samples were visualized through “ggplot2” package. To evaluate their diagnostic value, a column line graph was constructed based on the two crosstalk genes by using the “rms” package for clinical assessment of PD and SSc. Subsequently, receiver operating characteristic (ROC) curve analysis was conducted using the “pROC” package to assess the diagnostic performance of each gene. The area under the curve (AUC) was calculated to quantify diagnostic accuracy, AUC >0.7 was considered the ideal diagnostic value. All analyses were performed separately in discovery and validation cohorts for both PD and SSc.

### 2.8 Immune infiltration analysis

CIBERSORT employs a deconvolution algorithm to estimate the composition and abundance of 22 immune cell types from gene expression profiles. Immune cell infiltration analysis was performed using the “CIBERSORT” R package. The “ggplot2” package was used to visualize the relative proportions of immune cell subsets between disease and control groups. The correlation between hub gene and immune cells infiltration level were assessed using spearman’s correlation analysis, with a significance threshold set at *p* < 0.05. The results were visualized using the “ggplot2” R package.

### 2.9 Single-cell sequencing analysis

Single-cell RNA sequencing (scRNA-seq) data were processed using the Seurat R package (version 5.4.0). Low-quality cells were filtered based on the following criteria: nFeature_RNA >200, nCount_RNA >200, and percent. mt <25%. Data were log-normalized, and highly variable genes were identified for downstream analysis. Principal component analysis was performed, and batch effects across samples were corrected using the Harmony algorithm. A k-nearest neighbor graph was constructed, and cell clustering was conducted at a resolution of 0.9, with clusters visualized using uniform manifold approximation and projection (UMAP) and t-distributed stochastic neighbor embedding (t-SNE). Cell types were annotated based on canonical marker gene. Disease-associated gene identified from bulk transcriptomic analyses were examined across annotated cell populations, and differences in cell-type composition and gene expression between disease and control groups were further assessed. Gene expression levels shown in the plots were based on normalized expression values generated during the standard Seurat workflow. Single-cell datasets from periodontitis and systemic sclerosis were processed independently using the same analytical pipeline.

### 2.10 Prediction of transcription factors (TFs) and miRNAs

ENCODE, which provides extensive chromatin accessibility and transcription factor binding profiles derived from ChIP-seq experiments, was employed to predict transcription factors. MiRTarBase is a curated miRNA-target interactions database that included amounts of miRNAs and target genes supported by experimental evidence. The NetworkAnalyst 3.0 platform (https://www.networkanalyst.ca/), a comprehensive web-based visual analytics platform, was used to construct TF-gene and miRNA-gene regulatory network by integrating data from both databases. The final regulatory networks were visualized with Cytoscape software.

### 2.11 Screening of potential therapeutic drugs and molecular docking

The Drug Signatures Database (DSigDB) is a curated database integrating drug-gene annotations from multiple public sources (Yoo et al., 2015). It enables enrichment-based drug prediction analysis and has been widely used in drug repurposing studies. Based on these features, DSigDB was employed to identify potential therapeutic compounds targeting the common hub genes via the Enrichr platform (https://maayanlab.cloud/Enrichr/). Next, molecular docking was performed by CB-Dock2 (https://cadd.labshare.cn/cb-dock2/) to assess binding affinities and interaction patterns between candidate drugs and target proteins. The structural data of the candidate drugs were obtained from the PubChem database (https://pubchem.ncbi.nlm.nih.gov/) and converted into SDF format. The protein structural data were sourced from the UniProt database (https://www.uniprot.org/). According to previously reported criteria, an affinity value below −4.25 kcal/mol indicates binding activity between the components. Values lower than −5.0 kcal/mol suggest enhanced binding activity, while those under −7.0 kcal/mol point to robust docking activity between the two entities (Hsin et al., 2013).

### 2.12 Cell culture and transfection

Human periodontal ligament fibroblasts (HPDLFs) and human dermal fibroblasts (HDFs) were obtained from Procell (China). Cells were maintained in Dulbecco’s modified Eagle’s medium (DMEM, Gibco, USA) supplemented with 10% fetal bovine serum (FBS, Gibco, USA) and 1% penicillin-streptomycin (Gibco, USA) at 37 °C in a 5% CO_2_ incubator. To establish *in vitro* models of PD and SSc, HPDLFs were stimulated with 1 μg/mL of *Porphyromonas gingivalis* lipopolysaccharide (LPS, Sigma-Aldrich, USA) for 24 h, while HDFs were treated with 10 ng/mL of TGF-β1 (Peprotech, USA) for 24 h (Chang et al., 2025; Carvalheiro et al., 2020). For gene silencing experiments, cells were transfected with siRNAs targeting FNDC3B (siFNDC3B), NNMT (siNNMT), or a negative control siRNA (siNC) (Beijing Tsingke Biotech, China) using Lipofectamine 3000 (Invitrogen, USA) according to the manufacturer’s instructions.

### 2.13 CCK-8, Fe^2+^, and MDA level assays

Cell viability was assessed by the Cell Counting Kit-8 (Beyotime, China) according to the manufacturer’s instructions. Briefly, cells were seeded in 96-well plates at a density of 5 × 10^3^ cells per well. At the indicated time points (0, 24, 48, and 72 h), 10 µL CCK-8 was added to each well, followed by incubation for 4 h at 37 °C. Absorbance was subsequently measured at 450 nm using a microplate reader.

The cellular iron content was determined using Ferrous Ion Content Assay Kit (Solarbio, China). The malondialdehyde (MDA) levels were measured using Lipid Peroxidation MDA Assay Kit (Beyotime, China).

### 2.14 Quantitative real-time PCR

Total RNA was extracted using RNAex Pro Reagent (AG, China), and synthesized into cDNA with Evo M-MLV kit (AG, China). qRT-PCR was performed with AntiQ qPCR SYBR Green Fast Mix (Genesand, China) on a QuantStudio™ Real Time PCR System (Applied Biosystems, USA). β-actin was selected as an internal control for mRNA. Relative gene expression was calculated using the 2^−ΔΔCt^ method. The specific primer sequences used were shown in Supplementary Table S2.

### 2.15 Statistical analysis

All statistical analyses in this study were conducted using R software and GraphPad Prism. Experiment data were presented as mean ± standard deviation (SD). Group differences were assessed using the Wilcoxon rank-sum test and Student’s t-test, while correlations were evaluated by Spearman’s correlation analysis. A *p*-value less than 0.05 was considered statistically significant (^*^ *p* < 0.05, ^**^ *p* < 0.01, ^***^ *p* < 0.001, ^****^ *p* < 0.0001).

## 3 Results

### 3.1 Causal association between PD and SSc based on MR analysis

**FIGURE 2:**
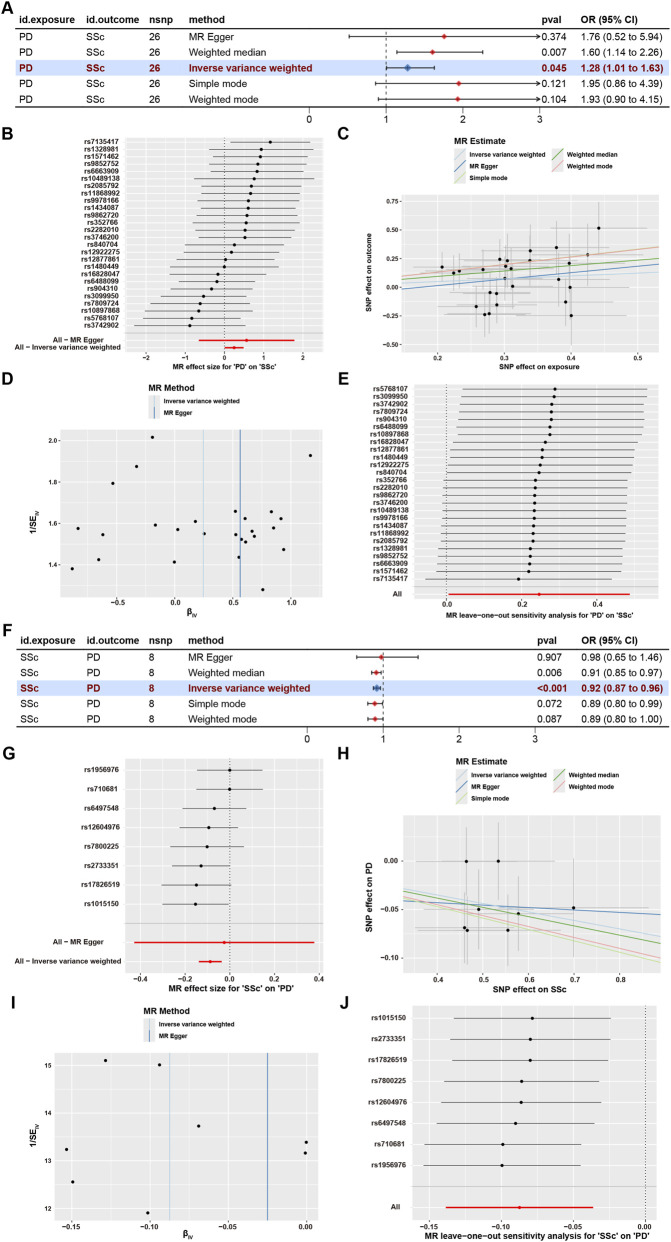
Bidirectional MR analysis between PD and SSc. **(A–E)** Forest plot, SNP forest plot, scatter plot, funnel plot and leave-one-out analysis illustrating the causal effect estimates of genetically predicted PD on SSc. **(F–J)** Forest plot, SNP forest plot, scatter plot, funnel plot, and leave-one-out analysis illustrating the causal effect estimates of genetically predicted SSc on PD.

To assess the potential causal relationship between PD and SSc, a bidirectional two-sample MR analysis was performed. In the forward analysis, a total of 26 SNPs associated with PD were selected as instrumental variables. The inverse variance weighted (IVW) method, used as the primary MR approach, suggested a possible association between genetically predicted PD and an increased risk of SSc (OR = 1.28, 95% CI = 1.01–1.63, P = 0.045), which was corroborated by the weighted median analysis (OR = 1.60, 95% CI = 1.14–2.26, P = 0.007). The remaining MR methods showed directionally consistent but nonsignificant estimates (Figures 2A–C). No significant heterogeneity (IVW: P = 0.555, MR-Egger: P = 0.513) and no evidence of pleiotropy (P = 0.608) were detected (Figure 2D; Supplementary Table S3). In addition, leave-one-out analysis indicated that the observed association was not driven by any single SNP (Figure 2E). In reverse analysis, 8 SSc-associated SNPs were selected as instrumental variables. Both the IVW (OR = 0.92, 95% CI = 0.87–0.96, P < 0.001) and weighted median (OR = 0.91, 95% CI = 0.85–0.97, P = 0.006) analyses indicated a potential protective association of SSc on PD (Figures 2F–H). No significant heterogeneity (IVW: P = 0.719, MR-Egger: P = 0.620) or pleiotropy (P = 0.770) was observed, and leave-one-out sensitivity analysis further supported the stability of the results (Figures 2I,J; Supplementary Table S3). Overall, the bidirectional MR analysis suggested a potential but asymmetric association between PD and SSc.

### 3.2 Identification of common ferroptosis-related DEGs (Co-FRDEGs)

**FIGURE 3:**
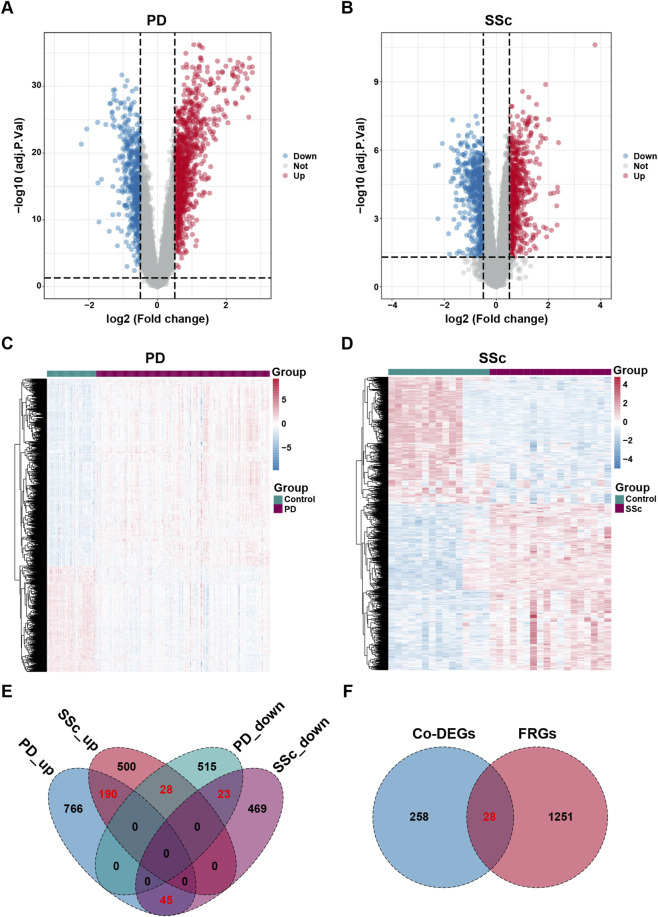
Identification of DEGs and Co-FRGs. **(A,B)** Volcano plot of all DEGs in PD (GSE16134) and SSc (GSE95065). **(C,D)** Clustered heatmap of all DEGs in PD (GSE16134) and SSc (GSE95065). **(E)** Venn diagram of all DEGs in PD (GSE16134) and SSc (GSE95065). **(F)** The intersection of Co-DEGs and FRGs.

Through differential expression analysis, a total of 1567 DEGs (including 1001 upregulated and 566 downregulated genes) were identified in the PD dataset GSE16134 (Figure 3A). Similarly, a total of 1255 DEGs (including 718 upregulated and 537 downregulated genes) were obtained from the SSc datasets GSE95065 (Figure 3B). The distribution and expression patterns of DEGs in both PD and SSc were visualized using volcano plots and heatmaps (Figures 3A–D). By intersecting the DEGs from the two datasets, 286 common DEGs (Co-DEGs) were identified (Figure 3E). We then cross-referenced Co-DEGs with a panel of 1279 ferroptosis-related genes (FRGs) from FerrDb V2 database, yielding 28 common ferroptosis-related DEGs (Co-FRDEGs) (Figure 3F).

### 3.3 Functional enrichment analysis of Co-FRDEGs

Analysis of GO and KEGG pathway enrichment were performed to explore the functions and pathways associated with the 28 Co-FRDEGs. A total of 345 GO terms were obtained, comprising 326 terms under the biological process (BP) category and 18 under molecular function (MF), while no significant enrichment was observed in the cellular component (CC) category. The top 10 enriched terms from each GO category were visualized (Supplementary Figure S1A). Regarding BP, most genes were predominantly enriched in taxis, chemotaxis, and response to lipopolysaccharide. For MF, most genes were mainly involved in cytokine activity, cytokine receptor binding, and organic anion transmembrane transporter activity. KEGG pathway analysis further revealed that these genes were significantly enriched in the NOD-like receptor signaling pathway, malaria, and IL-17 signaling pathway (Supplementary Figure S1B). The complete enrichment analysis results are listed in Supplementary Table S4. Taken together, these results strongly suggested that inflammatory and immune-related pathways may be shared pathologies in PD and SSc patients.

### 3.4 Weighted gene co-expression network analysis of PD and SSc

**FIGURE 4:**
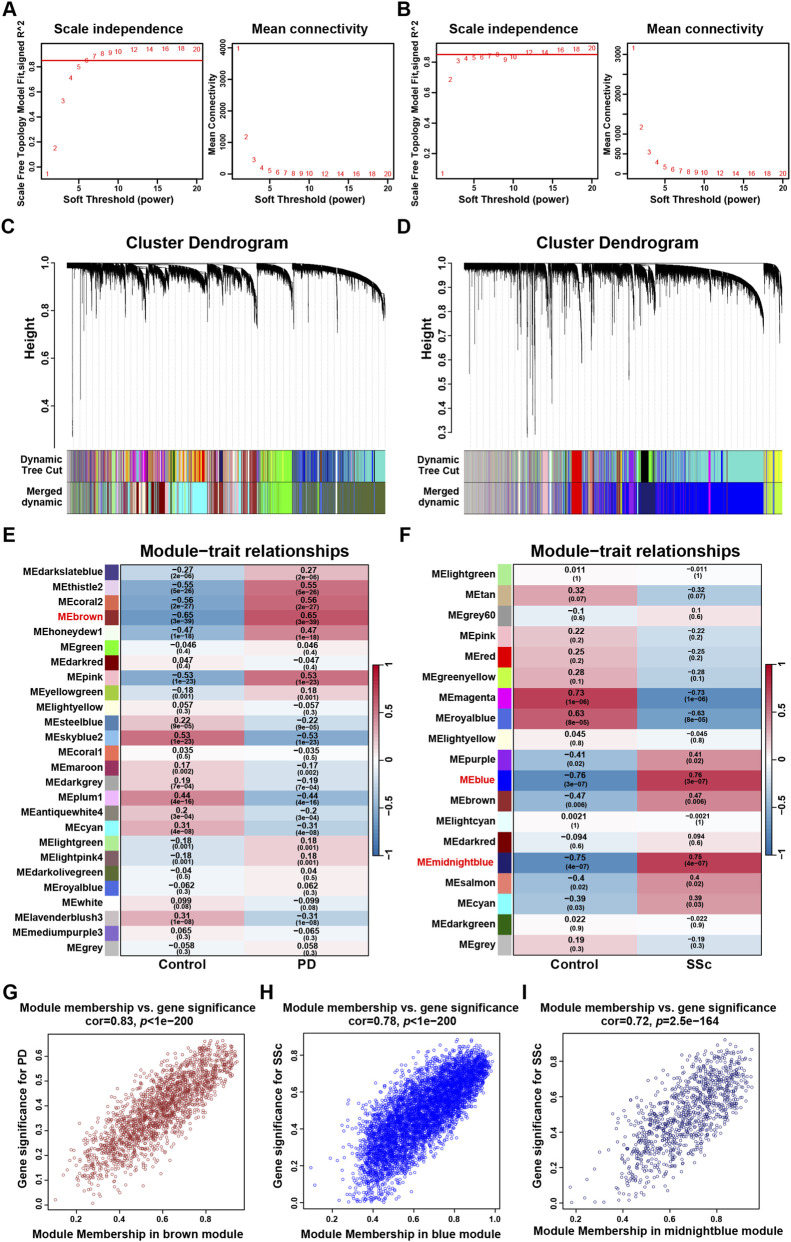
WCGNA analysis of PD and SSc. **(A,B)** Determination of the optimal soft thresholds for PD (GSE16134) and SSc (GSE95065). **(C,D)** The cluster dendrogram of co-expression genes in PD (GSE16134) and SSc (GSE95065). **(E,F)** Heatmap of the correlation analysis of ME with clinical phenotypes in PD (GSE16134) and SSc (GSE95065). **(G)** Scatter plot of gene significance for PD and module membership in the MEbrown module. **(H–I)** Scatter plot of gene significance for SSc and module membership in the MEblue and MEmidnightblue module.

WGCNA was performed to explore the most relevant module genes for disease. β = 7 for PD (GSE16134) and 12 for SSc (GSE95065) was selected as the soft-threshold power based on the scale independence and mean connectivity (Figures 4A,B). Subsequently, the cluster dendrogram was constructed separately and the similar gene modules were merged (Figures 4C,D). To evaluate the association between each gene module and disease status, a module-trait relationship heatmap was generated using Pearson’s correlation coefficients. In total, 26 co-expression modules were identified in the PD dataset, and 19 in the SSc dataset. Among them, the brown module (1833 genes, r = 0.65, *p* = 3e-39) exhibited a strong positive correlation with the occurrence of PD, while the blue module (5491 genes, r = 0.76, *p* = 3e-07) and midnightblue (1024 genes, r = 0.75, *p* = 4e-07) module were positively associated with the occurrence of SSc (Figures 4E,F). Furthermore, linear correlations between gene significance (GS) and module membership (MM) for the key modules were illustrated in scatter plots (Figures 4G,H). Lastly, genes from the key disease-associated modules were intersected with a curated list of FRGs using a Venn diagram, resulting in the identification of 63 common ferroptosis-related module genes (Co-FRMGs) (Supplementary Figure S2A).

### 3.5 Functional enrichment analysis of Co-FRMGs

GO and KEGG pathway enrichment analysis were also performed to uncover biological functions and signaling pathways associated with the 63 Co-FRMGs. A total of 514 GO terms were identified, including 485 BP terms and 28 MF terms, along with 13 KEGG pathways (detailed results were provided in Supplementary Table S5). The top 10 enriched terms in each category were visualized. As shown in Supplementary Figure S2B, BP terms mainly contained regulation of inflammatory response, cellular response to toxic substance, and reactive oxygen species metabolic process, while MF terms mainly included antioxidant activity, iron ion binding, and oxidoreductase activity (acting on peroxide as acceptor). KEGG pathway analysis was notably enriched in NOD-like receptor signaling pathway, lipid and atherosclerosis, and leishmaniasis (Supplementary Figure S2C). These results suggest that PD and SSc may share ferroptosis-related molecular mechanisms involving oxidative stress and immune-inflammatory regulation.

### 3.6 Screening of candidate diagnostic biomarkers via machine learning

**FIGURE 5:**
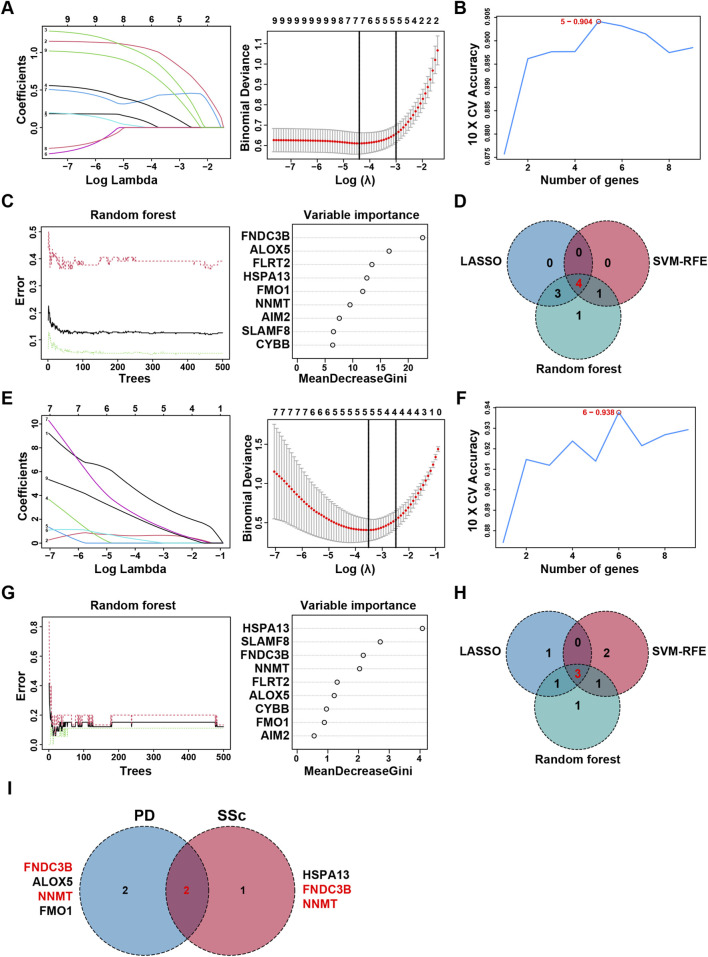
Identification of candidate diagnostic biomarkers by machine learning. **(A)** The LASSO regression model identified optimal lambda values and corresponding coefficients for diagnostic biomarkers in PD. **(B)** SVM-RFE analysis selected optimal feature genes with the highest classification accuracy for PD. **(C)** Random Forest ranked genes based on their importance scores across multiple decision trees in PD. **(D)** Venn diagram showing the overlapping genes obtained by three machine learning algorithms in PD. **(E)** The LASSO regression model identified optimal lambda values and corresponding coefficients for diagnostic biomarkers in SSc. **(F)** SVM-RFE analysis selected optimal feature genes with the highest classification accuracy for SSc. **(G)** Random Forest ranked genes based on their importance scores across multiple decision trees in SSc. **(H)** Venn diagram showing the overlapping genes obtained by three machine learning algorithms in SSc. **(I)** Venn diagram displaying intersecting diagnostic markers between PD and SSc.

To further explore key FRGs implicated in both PD and SSc, we intersected the previously obtained Co-FRDEGs and Co-FRMGs using a Venn diagram, which revealed 9 overlapping genes (Supplementary Figure S2D). These genes were subsequently analyzed using three independent machine learning algorithms (LASSO, SVM-RFE, and RF) to screen potential shared candidate genes with diagnostic relevance for the two diseases. For PD, following the 10-fold cross-validation procedure, LASSO regression selected seven diagnostic core genes (Figure 5A), while the SVM-RFE identified other five genes (Figure 5B). Concurrently, the RF algorithm ranked the genes based on the variable importance of each gene, with genes exhibiting a MeanDecreaseGini index greater than one deemed significant contributors (Figure 5C). By overlapping the results from the three algorithms, four overlapping genes (ALOX5, FMO1, FNDC3B, and NNMT) were ultimately suggested as potential diagnostic biomarkers for PD (Figure 5D). A similar analysis was applied to the SSc dataset. LASSO and SVM-RFE analyses identified five and six genes, respectively, following 10-fold cross-validation (Figures 5E,F). The RF algorithm identified six genes with high importance (Figure 5G). Cross-comparison of the results from these three algorithms revealed three overlapping genes (FNDC3B, HSPA13, and NNMT) with high diagnostic potential for SSc (Figure 5H). Ultimately, two genes (FNDC3B and NNMT) were suggested as shared candidate diagnostic markers for both PD and SSc after taking intersection (Figure 5I).

### 3.7 Diagnostic value assessment and validation of hub candidate diagnostic markers

**FIGURE 6:**
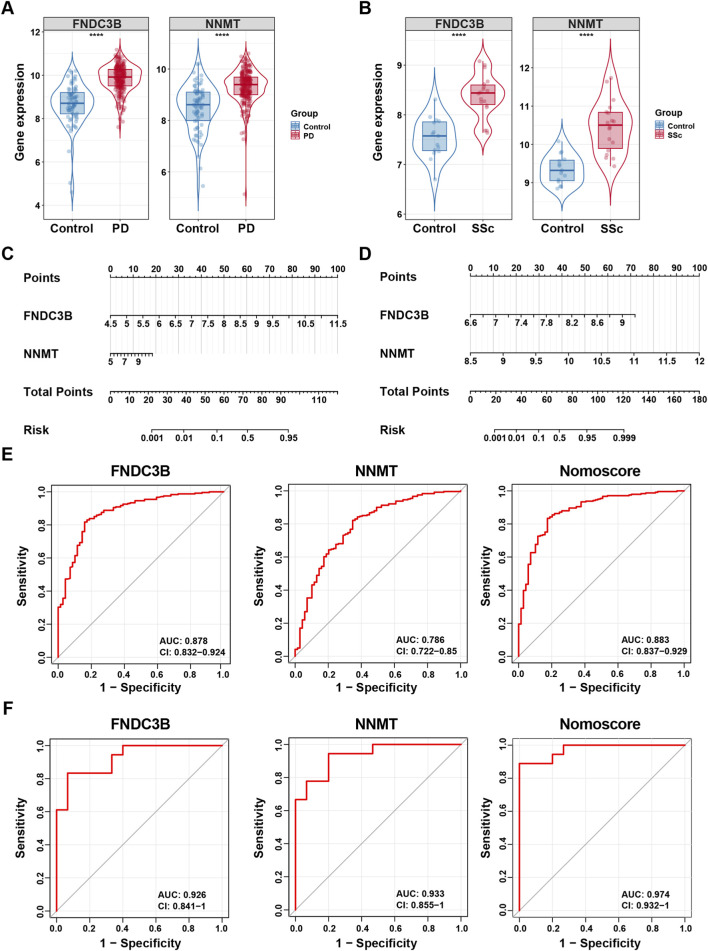
Nomogram construction and prediction accuracy evaluation in discovery datasets. **(A,B)** Differential expression analysis of two diagnostic biomarkers in discovery datasets for PD (GSE16134) and SSc (GSE95065). **(C,D)** Nomogram construction of two diagnostic biomarkers in discovery datasets for PD (GSE16134) and SSc (GSE95065). **(E,F)** ROC curves of two diagnostic biomarkers and nomoscore (a composite score derived from the two diagnostic biomarkers) in discovery datasets for PD (GSE16134) and SSc (GSE95065). ^****^ *p* < 0.0001.

To assess the diagnostic potential of FNDC3B and NNMT, we firstly investigated their expression levels in the PD and SSc discovery cohorts. The results showed that both genes were significantly upregulated in disease samples compared to controls (Figures 6A,B). Subsequently, a diagnostic nomogram incorporating both genes was developed to estimate the probability of disease (Figures 6C,D). Additionally, ROC analysis further ascertained their diagnostic utility. The results were as follows: FNDC3B (AUC: 0.878, 95%CI: 0.832–0.924), NNMT (AUC: 0.786, 95%CI: 0.722–0.85), and Nomoscore (AUC: 0.883, 95%CI 0.837–0.929) in PD discovery dataset (Figure 6E). FNDC3B (AUC: 0.926, 95%CI: 0.841–1), NNMT (AUC: 0.933, 95%CI: 0.855–1), and Nomoscore (AUC: 0.974, 95%CI 0.932–1) in SSc discovery dataset (Figure 6F). The AUC of all genes were larger than 0.75, indicating a good diagnostic value in both PD and SSc.

**FIGURE 7:**
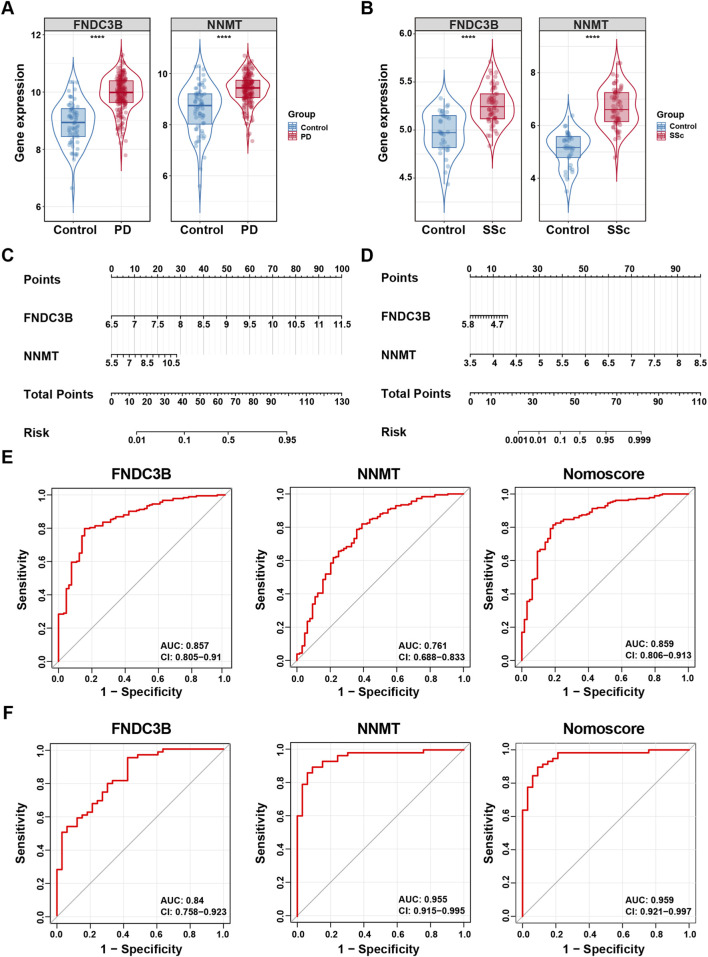
Nomogram construction and prediction accuracy evaluation in validation datasets. **(A,B)** Differential expression analysis of two diagnostic biomarkers in validation datasets for PD (GSE10334) and SSc (GSE130955). **(C,D)** Nomogram construction of two diagnostic biomarkers in validation datasets for PD (GSE10334) and SSc (GSE130955). **(E,F)** ROC curves of two diagnostic biomarkers and nomoscore (a composite score derived from the two diagnostic biomarkers) in validation datasets for PD (GSE10334) and SSc (GSE130955). ^****^ *p* < 0.0001.

To further verify the predictive performance of the two candidate hub genes, we validated them in two external datasets (GSE10334 for PD and GSE130955 for SSc). Consistent with the discovery cohorts, the expression levels of FNDC3B and NNMT were significantly elevated in disease group of validation datasets (Figures 7A,B). Moreover, diagnostic nomograms demonstrated consistent performance in validation cohorts, with all AUC values exceeding 0.75 (Figures 7C–F). In summary, the overall results suggested that FNDC3B and NNMT exhibited good discriminatory performance and may represent promising candidate shared biomarkers for both PD and SSc.

### 3.8 Analysis of immune cell infiltration

**FIGURE 8:**
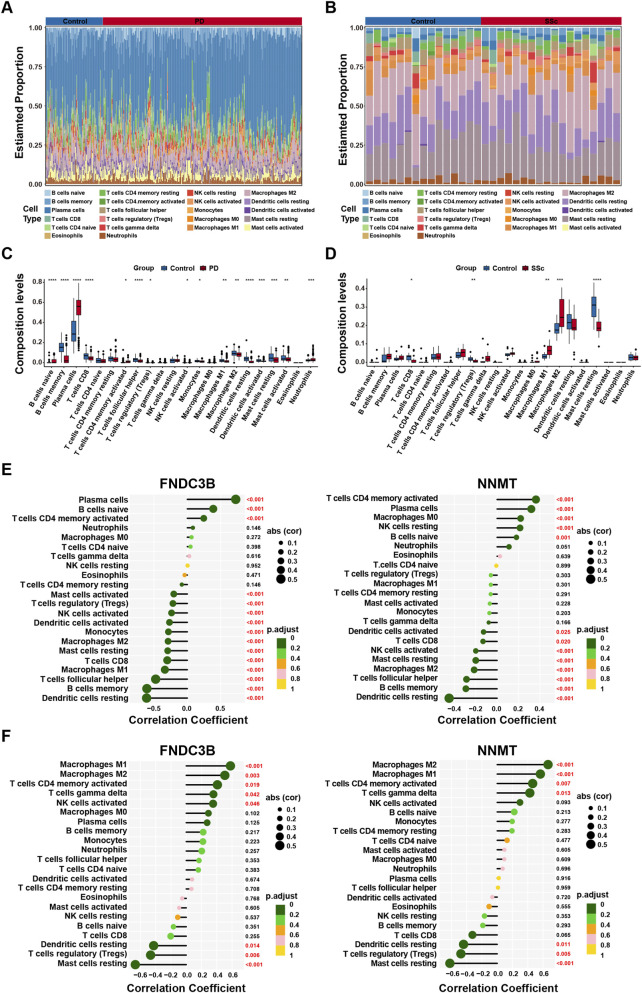
Immune cell infiltration analysis associated with PD and SSc. **(A,B)** Histogram of proportion of immune cells in different samples for PD and SSc. **(C,D)** Boxplot of the proportions of 22 infiltrating immune cells in different samples for PD and SSc. **(E,F)** Correlation analysis of infiltrating immune cells with two diagnostic markers in PD and SSc.

The initial enrichment analyses demonstrated that inflammatory and immune responses are vital for the development of both diseases, immune cell infiltration levels were assessed by CIBERSORT. The proportions of 22 immune cells in each sample from the disease and the control group were shown in Figures 8A,B. Compared to healthy group, the fractions of B cells naïve, plasma cells, T cells CD4 memory activated, and neutrophils were relatively greater in PD, while B cells memory, T cells CD8, T cells follicular helper, T cells regulatory (Tregs), NK cells activated, monocytes, macrophages M1, macrophages M2, dendritic cells resting, dendritic cells activated, mast cells resting, and mast cells activated were relatively less (Figure 8C). In SSc samples, notable upregulation was observed in macrophages M1 and macrophages M2, but lower levels of T cells CD8, T cells regulatory (Tregs) and mast cells resting (Figure 8D). Interestingly, both diseases shared decreased infiltration of T cells CD8, T cells regulatory (Tregs) and mast cells resting, suggesting involvement in a common pathogenic mechanism. Subsequently, the Spearman correlation analysis between candidate diagnostic biomarkers and various immune cells were performed. The results indicated that both FNDC3B and NNMT were negatively correlated with dendritic cells resting and mast cells resting in PD and SSc samples (Figures 8E,F). These findings imply that these genes may play crucial roles in the pathogenesis of PD complicated by SSc via modulation of the local immune microenvironment.

### 3.9 Expression of hub candidate diagnostic markers in single cell level

**FIGURE 9:**
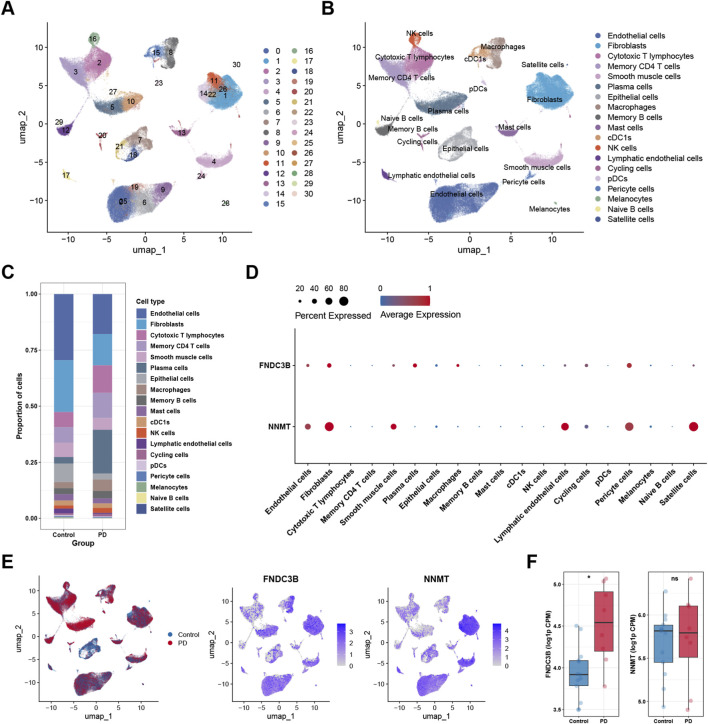
scRNA-seq analysis in PD. **(A)** UMAP plot of cell clustering. **(B)** UMAP plot of different cell types after cell annotation. **(C)** The proportion of each cell cluster in control and PD samples. **(D)** Bubble plot showing the expression levels and proportions of hub diagnostic genes across different cell types. **(E)** UMAP feature plots showing the expression distribution of hub diagnostic genes across different cell types. **(F)** Violin plots depicting the expression of hub diagnostic genes in control and PD samples. ^*^ *p* < 0.05.

**FIGURE 10:**
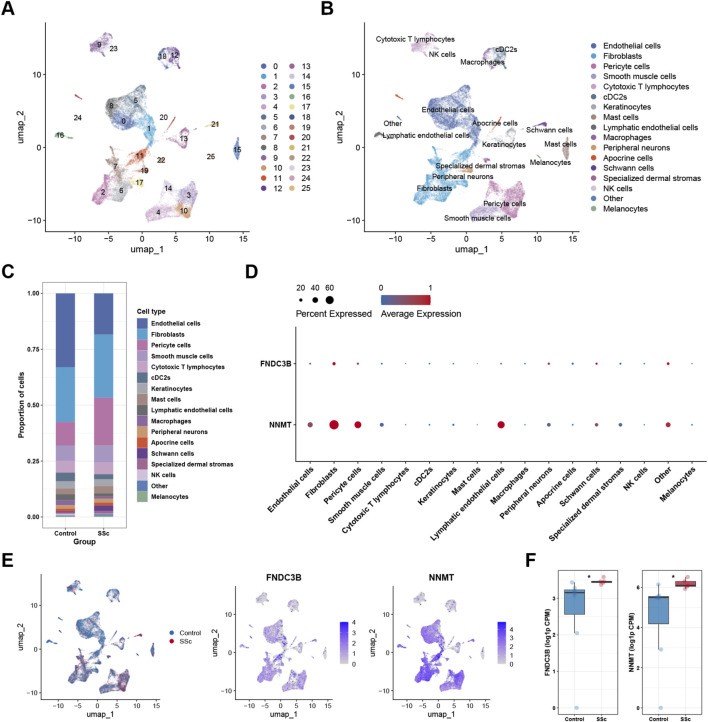
scRNA-seq analysis in SSc. **(A)** UMAP plot of cell clustering. **(B)** UMAP plot of different cell types after cell annotation. **(C)** The proportion of each cell cluster in control and SSc samples. **(D)** Bubble plot showing the expression levels and proportions of hub diagnostic genes across different cell types. **(E)** UMAP feature plots showing the expression distribution of hub diagnostic genes across different cell types. **(F)** Violin plots depicting the expression of hub diagnostic genes in control and SSc samples. ^*^ *p* < 0.05.

To further elucidate the cellular basis underlying the immune infiltration patterns identified by bulk transcriptomic analysis, scRNA-seq was employed to delineate cell type-specific expression patterns of the two candidate diagnostic markers in PD and SSc. Two independent scRNA-seq datasets were analyzed, including GSE164241 for PD and GSE214088 for SSc. Following quality control and batch correction, a total of 94,783 cells were retained and classified into 30 clusters in the PD dataset, whereas 25,509 cells were grouped into 25 clusters in the SSc dataset (Figures 9A, 10A). Depending on the expression of canonical marker genes, these clusters were subsequently annotated 19 distinct cell types for PD and 17 cell types for SSc (Figures 9B, 10B). The relative proportions of each cell type in control and disease samples are shown in Figures 9C, 10C. Next, we assessed the expression levels of two hub markers (FNDC3B and NNMT) of all the cell types in PD and SSc, respectively. In PD, FNDC3B expression was predominantly enriched in fibroblasts, plasma cells, and pericyte cells, while NNMT showed higher expression levels in endothelial cells, fibroblasts, smooth muscle cells, pericyte cells, and satellite cells (Figures 9D,E). In the context of SSc, FNDC3B was broadly expressed in fibroblasts, whereas NNMT expression was mainly localized to endothelial cells, fibroblasts, and pericyte cells (Figures 10D,E). Notably, the overall cellular distribution patterns of these two hub markers were largely conserved between PD and SSc, indicating shared cell type-specific expression characteristics across the two diseases. Furthermore, FNDC3B was upregulated in both diseases, whereas NNMT was elevated only in SSc (Figures 9F, 10F). Collectively, these single-cell findings provide cellular-level evidence that links the immune infiltration alterations observed in bulk transcriptomic analyses to specific cell populations, thereby offering mechanistic insight into the shared and disease-specific immune features of PD and SSc.

### 3.10 Construction of TFs, miRNAs and shared markers interaction network

**FIGURE 11:**
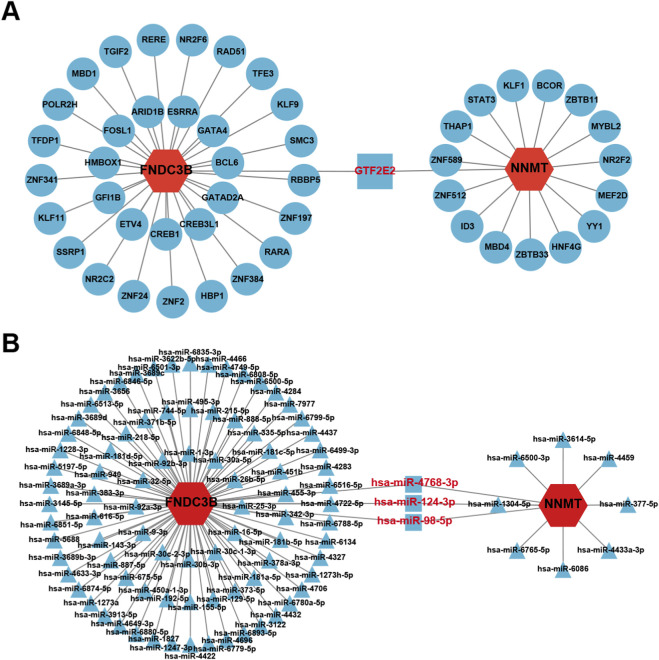
The regulatory network of shared markers with TFs and miRNAs. **(A)** TF-gene regulatory network of two shared markers. **(B)** miRNA-gene regulatory network of two shared markers.

To explore potential transcriptional and post-transcriptional regulators of the shared markers, the NetworkAnalyst platform was used to search for associated TFs and miRNAs that may regulate their expression. The TF-gene interaction network was identified by ENCODE databases, including 50 nodes and 49 edges (Figure 11A). Similarly, the miRNA-gene interaction network was established using miRTarBase, consisting of 99 nodes and 100 edges (Figure 11B). These prediction results were visualized using Cytoscape. Notably, GTF2E2 and three miRNAs (hsa-miR-124-3p, hsa-miR-4768-3p, and hsa-miR-98-5p) respectively interacted with two shared markers simultaneously, suggesting their potential as common regulatory factors influencing PD and SSc pathogenesis. However, these findings require further experimental validation.

### 3.11 Prediction of potential lead compounds and molecular docking

Potential therapeutic compounds targeting shared candidate biomarkers were screened using the Drug Signatures database (DSigDB) within the enrichment platform (Supplementary Table S6). Candidate drugs were ranked by *P*-values and adjusted *P*-values, with the top 10 candidates comprising: Thapsigargin, Diflorasone, Withaferin A, Simvastatin, Isoflupredone, Ionomycin, Geldanamycin, Pyrvinium, Mometasone, and Troglitazone (Table 2). Among them, only thapsigargin is related to NNMT and FNDC3B with the highest combined score, indicating it as a potential lead compound for further investigation.

**TABLE 2:** Predicted top 10 drug compounds.

Term	P-value	Adjusted P-value	Combined score	Genes
Thapsigargin	0.000584	0.041596	290587.4	NNMT, FNDC3B
Diflorasone	0.001999	0.041596	6535.343	NNMT
Withaferin A	0.002898	0.041596	4167.854	NNMT
Simvastatin	0.003197	0.041596	3700.591	NNMT
Isoflupredone	0.004395	0.041596	2518.618	NNMT
Ionomycin	0.004495	0.041596	2451.073	FNDC3B
Geldanamycin	0.004595	0.041596	2386.751	NNMT
Pyrvinium	0.00529	0.041596	2010.466	NNMT
Mometasone	0.005991	0.041596	1729.450	NNMT
Troglitazone	0.006190	0.041596	1661.878	NNMT

**FIGURE 12:**
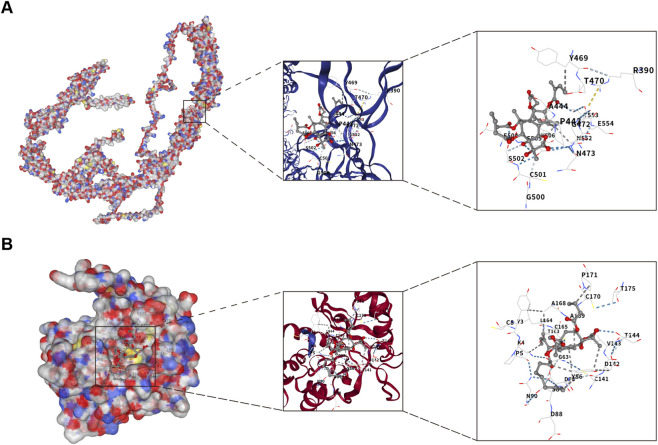
Molecular docking results for drug and target proteins. **(A)** FNDC3B docking thapsigargin. **(B)** NNMT docking thapsigargin.

To further understand the affinity between drugs and target proteins, and to gain insights into the druggability of these targets, we conducted molecular docking studies. Thapsigargin displayed binding energy of −6.3 kcal/mol with FNDC3B and −6.1 kcal/mol with NNMT (Table 3), suggesting good binding activity. Interaction models were visualized using CB-Dock2 (Figure 12). Therefore, thapsigargin may serve as a potential lead compound identified through computational screening for PD-SSc comorbidity warranting further investigation.

**TABLE 3:** Molecular docking results for drug and target proteins.

Target	Identifier	Drug	PubChem ID	Binding energy (kcal/mol)
FNDC3B	7EI2	Thapsigargin	446378	−6.3
NNMT	AF-Q53EP0-F1			−6.1

### 3.12 Candidate biomarkers expression and function validation

**FIGURE 13:**
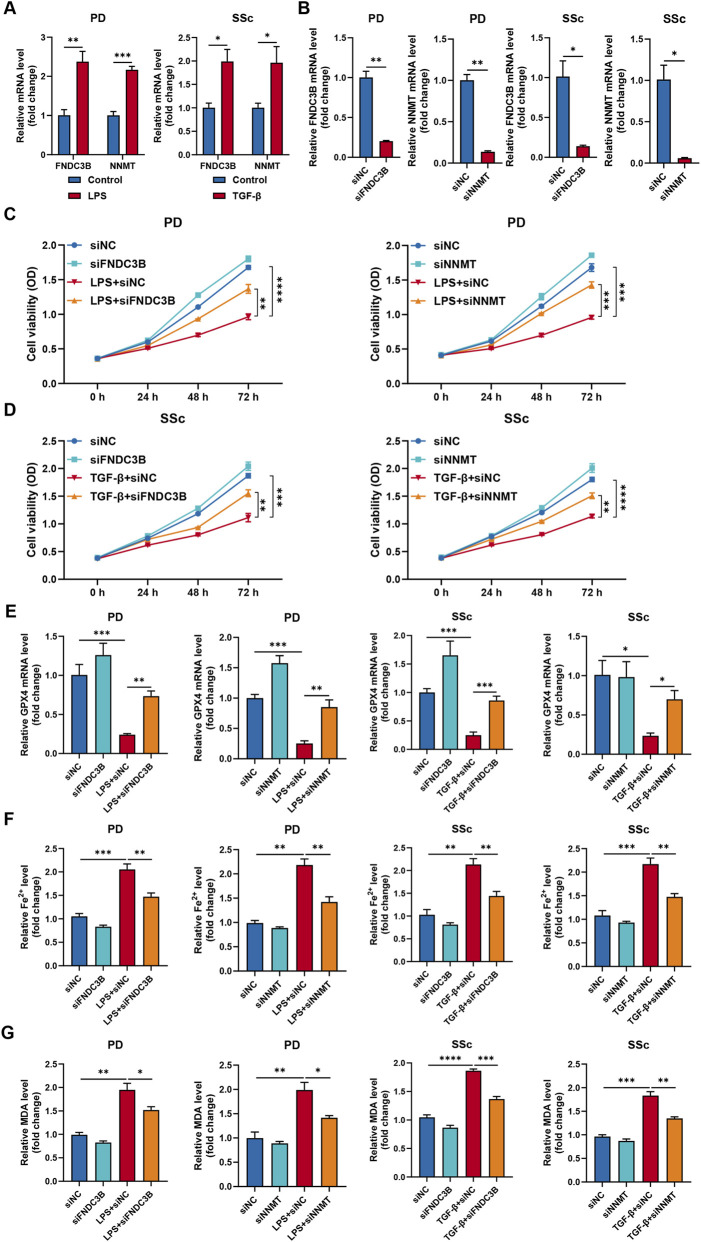
Functional validation of FNDC3B and NNMT in ferroptosis regulation in PD and SSc cell models. **(A)** Relative mRNA expression levels of FNDC3B and NNMT in LPS-treated HPDLFs and TGF-β-treated HDFs compared with control groups. **(B)** Knockdown efficiency of FNDC3B and NNMT in HPDLFs and HDFs following siRNA transfection. **(C,D)** Cell viability assessed by CCK-8 assay in LPS-treated HPDLFs and TGF-β-treated HDFs following FNDC3B or NNMT knockdown. **(E)** Relative mRNA expression levels of GPX4 in LPS-treated HPDLFs and TGF-β-treated HDFs following FNDC3B or NNMT knockdown. **(F,G)** Quantification of Fe^2+^ and MDA levels in LPS-treated HPDLFs and TGF-β-treated HDFs following FNDC3B or NNMT knockdown. ^**^ *p* < 0.05, ^**^ *p* < 0.01, ^***^ *p* < 0.001, ^****^ *p* < 0.0001.

To validate the bioinformatic findings, *in vitro* models of PD and SSc were established using HPDLFs and HDFs to mimic inflammatory conditions. The results demonstrated the expression levels of FNDC3B and NNMT were significantly upregulated in the disease-mimicking groups compared with controls (Figure 13A). To further investigate the roles of FNDC3B and NNMT in ferroptosis within PD and SSc contexts, gene silencing experiments were performed in HPDLFs and HDFs. Knockdown efficiency was confirmed by qRT-PCR (Figure 13B). Subsequently, key ferroptosis-associated markers were evaluated. LPS-treated HPDLFs and TGF-β-treated HDFs exhibited reduced cell viability and decreased GPX4 expression, accompanied by elevated intracellular Fe^2+^ accumulation and increased MDA levels, indicating activation of ferroptotic processes (Figures 13C–G). Notably, silencing of FNDC3B or NNMT significantly partially restored cell viability and GPX4 expression, while reducing Fe^2+^ and MDA levels. Collectively, these results suggest that FNDC3B and NNMT may contribute to PD- and SSc-related cellular injury by promoting ferroptosis, highlighting their potential roles in disease pathogenesis.

## 4 Discussions

PD and SSc are both chronic inflammatory diseases that profoundly impact human health and quality of life. Prior research has demonstrated that PD and SSc act as mutual risk factors, exhibiting a bidirectional association (Sredojevic et al., 2024). Additionally, cytokines such as TGF-β1 and VEGF have been reported to participate in immune regulation and vascular dysfunction in both conditions, offering a potential mechanistic link (Matarese et al., 2012). A recent study further implicated that oxidative stress is a shared pathogenic driver contributing to immune dysregulation and tissue damage in PD and SSc (Sredojevic et al., 2023). Given that ferroptosis is characterized by iron-dependent lipid peroxidation and redox imbalance, we hypothesized that ferroptosis may contribute to the comorbidity between PD and SSc. However, the involvement of ferroptosis in PD complicated by SSc has not been systematically explored. Thus, this study aimed to elucidate the common FRGs and mechanisms linking PD and SSc and to identify potential diagnostic and therapeutic targets using integrated bioinformatics.

As an initial step, bidirectional MR analysis was performed to assess whether the reported comorbidity between PD and SSc reflects a potential causal relationship. The forward analysis suggested that genetically predicted PD was associated with an increased risk of SSc, which is consistent with the hypothesis that chronic periodontal inflammation may contribute to systemic autoimmune pathology. Conversely, the reverse MR analysis showed a potential protective association of SSc on PD. Given the limited statistical power of the available GWAS datasets and the possibility of weak instrument bias, this unexpected result may reflect statistical fluctuation rather than a true biological protective effect. Overall, considering the relatively small GWAS sample sizes and the use of a relaxed SNP selection threshold, the MR findings should be regarded as preliminary and hypothesis-generating, rather than definitive evidence of causality. Future studies using larger and better-powered GWAS datasets will be necessary to validate and clarify the potential causal relationship between PD and SSc.

To comprehensively capture FRGs associated with PD-SSc comorbidity, we combined DEGs and WGCNA analysis, yielding 28 Co-FRDEGs and 63 Co-FRMGs between PD and SSc, respectively. These candidate genes were mainly enriched in inflammatory and immune response, oxidative stress, regulation of signaling pathways, and infectious disease, consistent with previous reports that both PD and SSc share inflammatory and immune dysregulation. Elevated pro-inflammatory cytokines, including TNF-α, IL-6, IL-1, and IL-17, are present in both diseases (Cüzdan et al., 2021). Clinically, both diseases severely compromise oral health, leading to gingival microvascular injury, sustained inflammation, alveolar bone resorption, and loosening of teeth (Elimelech et al., 2015). Mechanistically, excessive reactive oxygen species (ROS) production plays a critical role in periodontal tissue destruction and impaired healing in PD (Shang et al., 2023), while oxidative stress in SSc contributes to endothelial dysfunction, fibroblast activation, and progressive tissue fibrosis (Abdulle et al., 2018). Notably, altered antioxidant enzyme activities, including elevated GPX and SOD levels, have been reported in patients with concomitant PD and SSc, further supporting oxidative stress as a shared pathogenic axis (Sredojevic et al., 2023). Ferroptosis, which is tightly linked to lipid peroxidation and redox imbalance, may therefore represent a critical mechanistic bridge between the two diseases. Furthermore, environmental triggers such as infections and toxic exposures may further exacerbate immune dysregulation and inflammation in both diseases (Ciurea et al., 2023).

Intersecting Co-FRDEGs and Co-FRMGs identified nine shared genes. Using three machine-learning algorithms (LASSO, SVM-RFE, and random forest), we further narrowed these to two hub candidate diagnostic genes: FNDC3B and NNMT. Both were significantly upregulated in PD and SSc and exhibited good diagnostic performance (AUC >0.75) across discovery and validation cohorts, highlighting their potential roles in the pathogenesis and diagnosis of PD-SSc comorbidity.

Fibronectin type III domain containing 3B (FNDC3B, also known as FAD104), initially emerged as a regulator of adipocyte and osteoblast differentiation (Katoh et al., 2016). Increasing evidence indicates that FNDC3B functions as an oncogenic driver in several cancers, including hepatocellular, nasopharyngeal, and oral tongue squamous cell carcinoma (Lin et al., 2016; Li et al., 2020; Zhong et al., 2018). Yang et al. found that FNDC3B attenuated ferroptosis of OSCC cells by regulating miR-520d-5p/SLC7A11 axis to promote OSCC progression (Yang et al., 2021). Similarly, *FNDC3B* was upregulated in alcoholic liver disease, where it mitigated ferroptosis through modulation of lipid and iron metabolism (You et al., 2022). However, elevated FNDC3B expression enhanced angiotensin II (Ang-II)-triggered cytotoxicity in human aortic VSMCs by promoting oxidative stress and inflammation (Liu et al., 2021). It also suppressed hepatocellular carcinoma proliferation by disrupting SREBP-dependent lipid metabolism and inducing oxidative stress (Feng et al., 2017). Consistent with these findings, our results showed FNDC3B upregulation in both PD and SSc, enriched in inflammatory and oxidative stress pathways. Moreover, our *in vitro* experiments showed that silencing FNDC3B partially reversed ferroptosis-related changes, including reduced GPX4 expression and increased Fe^2+^ and MDA levels in disease-mimicking cellular models. These results suggest that FNDC3B might participate in the co-pathogenesis of PD and SSc through ferroptosis-associated mechanisms. However, given its context-dependent roles across diseases, further studies are required to clarify the precise regulatory mechanisms of FNDC3B in PD and SSc.

Nicotinamide N-methyltransferase (NNMT) is an epigenetic enzyme that catalyzes N-methylation of nicotinamide using S-adenosylmethionine to generate 1-methylnicotinamide and S-adenosyl-L-homocysteine (Alston and Abeles, 1988). NNMT is extensively expression in the liver and adipose tissue and correlation with the pathogenesis of several diseases including cancer, neurodegenerative diseases, cardiovascular diseases, and metabolic diseases (Li et al., 2024). Recent studies revealed that NNMT prompted ferroptosis via inhibiting SLC7A11/GPX4 in deoxynivalenol-induced hepatocyte toxicity (Huang et al., 2025). Bioinformatics analyses have also linked NNMT-associated ferroptosis to pathological conditions, such as renal carcinoma, lupus nephritis, and ischemic cardiomyopathy (Pang et al., 2024; Xiao et al., 2025; Guo et al., 2024). In our analysis, NNMT emerged as a potential diagnostic biomarker contributing to PD and SSc pathogenesis. The additional *in vitro* experiments further supported its involvement in ferroptosis-associated cellular responses under disease-mimicking conditions. Given the availability of NNMT inhibitors, targeting this enzyme may represent a novel therapeutic approach, though additional experimental validation is required.

Our enrichment analysis indicated that immune dysregulation is a common feature in both PD and SSc. To further characterize the immune landscape, we performed CIBERSORT analysis and identified coordinated alterations across several immune cell subsets, including reduced proportions of CD8^+^ T cells, regulatory T cells (Tregs), and resting mast cells. Previous studies have reported inconsistent findings regarding CD8^+^ T cell and mast cell infiltration in PD, possibly due to variations in sample type and analytical methods (Nagasawa et al., 1995; Cifcibasi et al., 2015; Okui et al., 2014; Fattahi et al., 2019). Functionally, Tregs are essential for maintaining immune tolerance and periodontal homeostasis through the secretion of anti-inflammatory cytokines such as TGF-β and IL-10 (Cardoso et al., 2008). Therefore, impairment of Tregs function may exacerbate PD. In SSc, immune dynamics are known to be stage-dependent, with CD8^+^ T cells predominating in early disease and CD4^+^ T cells becoming more prominent during fibrotic progression (Medsger, 2003; Fuschiotti et al., 2013). Although reports regarding Treg abundance in SSc remain conflicting, most studies indicate that Tregs exhibit functional defects regardless of their frequency (Slobodin and Rimar, 2017). Mast cells are also implicated in early SSc pathogenesis through the release of pro-fibrotic mediators such as histamine, tryptase, and TGF-β (Fang et al., 2022). Interestingly, our analysis revealed diminished mast cell infiltration in SSc group, which may reflect functional exhaustion of these cells following sustained activation in fibrotic progression.

Importantly, these immune alterations may be mechanistically linked to the two key candidates predicted in our study. FNDC3B has been implicated in extracellular matrix organization and stromal remodeling, processes that critically regulate cytokine gradients, t issue architecture, and immune cell recruitment (Khanum et al., 2026; Di Vito et al., 2024). In inflammatory and fibrotic settings such as PD and SSc, aberrant FNDC3B expression within stromal compartments may indirectly contribute to influence CD8^+^ T cell surveillance and immune regulation. NNMT, in contrast, functions as a metabolism-associated regulator that reshapes methyl-donor availability and redox balance (Xie et al., 2020). Because oxidative stress and ferroptosis are closely intertwined with immune regulation, elevated NNMT may promote a redox-imbalanced microenvironment that influences immune cell composition and function (Chen et al., 2021). Together, these findings suggest a potential a stromal-metabolic-immune axis linking FNDC3B and NNMT dysregulation to the convergent immune infiltration patterns observed in PD and SSc. Nevertheless, these mechanistic links remain speculative and require further experimental validation.

To determine whether this proposed indirect regulatory model is supported at the cellular level, we performed scRNA-seq analysis to define the cell-type specificity of FNDC3B and NNMT expression. In both PD and SSc, FNDC3B was predominantly expressed in fibroblasts and stromal-related cell populations, whereas NNMT was enriched in endothelial cells, fibroblasts, pericytes, and smooth muscle-associated cells. These conserved cellular expression patterns across the two diseases suggest that FNDC3B and NNMT primarily act within stromal and vascular compartments rather than immune cells themselves. Notably, while NNMT expression was upregulated in bulk PD samples, a significant increase was not observed at the single-cell level in PD, which may be attributable to differences in dataset composition, cellular heterogeneity, and technical characteristics inherent to bulk and single-cell sequencing approaches. Overall, these observations suggest that alterations in stromal and endothelial metabolic states and redox balance may indirectly influence the immune microenvironment, potentially contributing to the immune alterations observed in PD and SSc.

It is well known that TFs regulate target gene expression, whereas miRNAs modulate post-transcriptional regulation. Regulatory network analysis predicted GTF2E2 and three miRNAs (hsa-miR-124-3p, hsa-miR-4768-3p, and hsa-miR-98-5p) as potential coregulators of both biomarkers. Transcription initiation factor IIE subunit beta (GTF2E2), an essential component of the RNA polymerase II transcription initiation complex, is involved in diverse biological processes, including stem cell regulation and viral replication (Theil et al., 2019; Liu et al., 2023; Dziuba et al., 2012). Abnormal GTF2E2 expression has been associated with poor cancer prognosis, and its knockdown was shown to induce ferroptosis via modulation of GPX4 and ACSL4 in uterine carcinoma (Zhang et al., 2025). hsa-miR-124-3p negatively regulates inflammation and ferroptosis in multiple diseases (Wu et al., 2022; Zhang et al., 2022; Xie et al., 2023). While research on hsa-miR-4768-3p remains limited, it associates with progression and metastasis of osteosarcoma (Zheng et al., 2023). hsa-miR-98-5p is implicated in antioxidant defense mechanisms across various diseases and functions as a tumor suppressor in multiple tumors (Guo et al., 2023; Zhang K. et al., 2024). Additionally, Tan et al. revealed that the LINC02432/hsa-miR-98-5p/HK2 axis, associated with glycolytic regulation, inhibited ferroptosis in pancreatic adenocarcinoma through bioinformatics analysis (Tan et al., 2022). Collectively, these previous findings suggest that the predicted TFs and miRNAs may be associated with ferroptosis, raising the possibility that they could participate in regulating FNDC3B and NNMT expression in the context of comorbid PD and SSc. However, it should be noted that the regulatory interactions identified in this study are based on computational prediction and require further experimental validation, such as ChIP-qPCR or luciferase reporter assays, to confirm direct regulatory relationships.

Furthermore, we utilized the DSigDB database to identify the top 10 potential therapeutic compounds targeting shared genes. Among them, thapsigargin uniquely targeted both shared biomarkers. Molecular docking analysis further confirmed good binding affinity between thapsigargin and these shared targets. Thapsigargin, a sesquiterpene lactone isolated from *Thapsia garganica*, has a long history of use in traditional medicine for treating rheumatic pain, pulmonary diseases, and female infertility (Andersen et al., 2015). Its primary mechanism involves inhibition of sarcoplasmic/endoplasmic reticulum calcium ATPase (SERCA), thereby inducing endoplasmic reticulum stress and cell death in various cancers (Jaskulska et al., 2020). Wang et al. demonstrated that thapsigargin inhibited MH7A cell proliferation via mTOR-cyclin D1 pathway, suggesting potential therapeutic utility in rheumatoid arthritis (Wang et al., 2014). It also alleviates TNF-α-induced NF-κB activation by promoting p38 MAPK signaling, indicating potential anti-inflammatory effects in inflammatory bowel disease (Uwada et al., 2019). Notably, thapsigargin attenuates inflammation in human gingival epithelial cells and inhibits *Porphyromonas gingivalis* invasion by reducing intracellular calcium release (Izutsu et al., 1996; Son et al., 2016). So, thapsigargin may hold some potential therapeutic value in PD and SSc. The function and mechanism of action can be further investigated in the future to provide a reference for clinical use.

Despite the promising findings, this study has several limitations. First, due to limited sample sizes of datasets and the unavailability of public comorbidity datasets for PD and SSc, larger cohorts and comorbidity datasets are needed for future validation. Second, the variety of sample types and detection microarray platforms resulted in bias that needed more datasets to verify our findings. Third, although TF-miRNA regulatory networks and potential therapeutic compounds were predicted through database-based analyses, these results remain computational predictions and require further experimental validation.

## 5 Conclusion

This study systematically explored ferroptosis-related genes and regulatory mechanisms between PD and SSc by using integrated bioinformatics and experimental validation. Bidirectional MR analysis suggested a potential association between PD and SSc, providing preliminary genetic evidence for their comorbidity. FNDC3B and NNMT were predicted as candidate shared biomarkers, and preliminary *in vitro* experiments supported their potential involvement in ferroptosis-related cellular responses under disease-mimicking conditions. Inflammation and immune responses pathways also appear central to the shared pathogenesis. In summary, this study shows significant implications for understanding the pathogenesis of two diseases and provides new biological targets and ideas for diagnosis and effective treatment of PD combined with SSc.

## Data Availability

The original contributions presented in the study are included in the article/Supplementary Material, further inquiries can be directed to the corresponding authors.
