# Supplementary material for: Exploring the common ferroptosis-related genes and molecular mechanisms in periodontitis and systemic sclerosis via integrated bioinformatics and experimental analysis
Source: Front Cell Dev Biol. 2026 Apr 2;14:1803091. doi: 10.3389/fcell.2026.1803091 (PMC13083135; doi:10.3389/fcell.2026.1803091)
Supplement: Supplementary file 3 [file Table2.docx]

**Supplementary Table S2** Primer sequences used in this study.

| Primer name | Sequence (5’-3’) |
| --- | --- |
| qPCR-β-actin-F | CACCATTGGCAATGAGCGGTTC |
| qPCR-β-actin-R | AGGTCTTTGCGGATGTCCACGT |
| qPCR-FNDC3B-F | CCACCTGTTACCGGACCTG |
| qPCR-FNDC3B-R | GGGTGATGTAGGTTGACATTCC |
| qPCR-NNMT-F | GAGATCGTCGTCACTGACTACT |
| qPCR-NNMT-R | CACACACATAGGTCACCACTG |
| qPCR-GPX4-F | GAGGCAAGACCGAAGTAAACTAC |
| qPCR-GPX4-R | CCGAACTGGTTACACGGGAA |
