# Supplementary material for: Exploring the common ferroptosis-related genes and molecular mechanisms in periodontitis and systemic sclerosis via integrated bioinformatics and experimental analysis
Source: Front Cell Dev Biol. 2026 Apr 2;14:1803091. doi: 10.3389/fcell.2026.1803091 (PMC13083135; doi:10.3389/fcell.2026.1803091)
Supplement: Supplementary file 7 [file DataSheet1.docx]

**Supplementary S1**


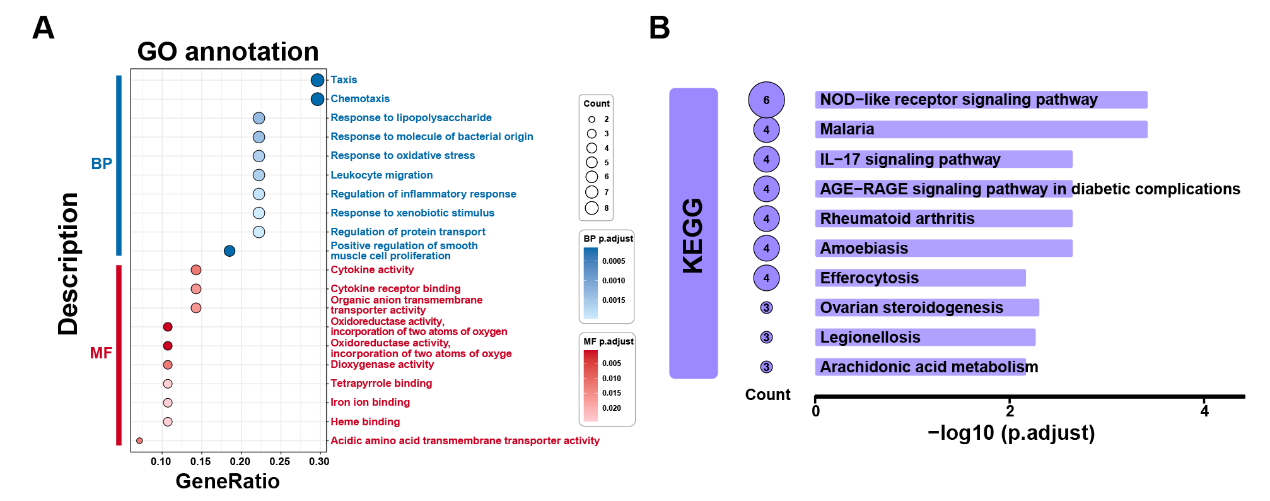


**Functional enrichment analysis of Co-FRDEGs. (A)** Bubble plots of GO enrichment analysis results for BP and MF. **(B)** Bar plots of KEGG pathway enrichment analysis.

**Supplementary S2**


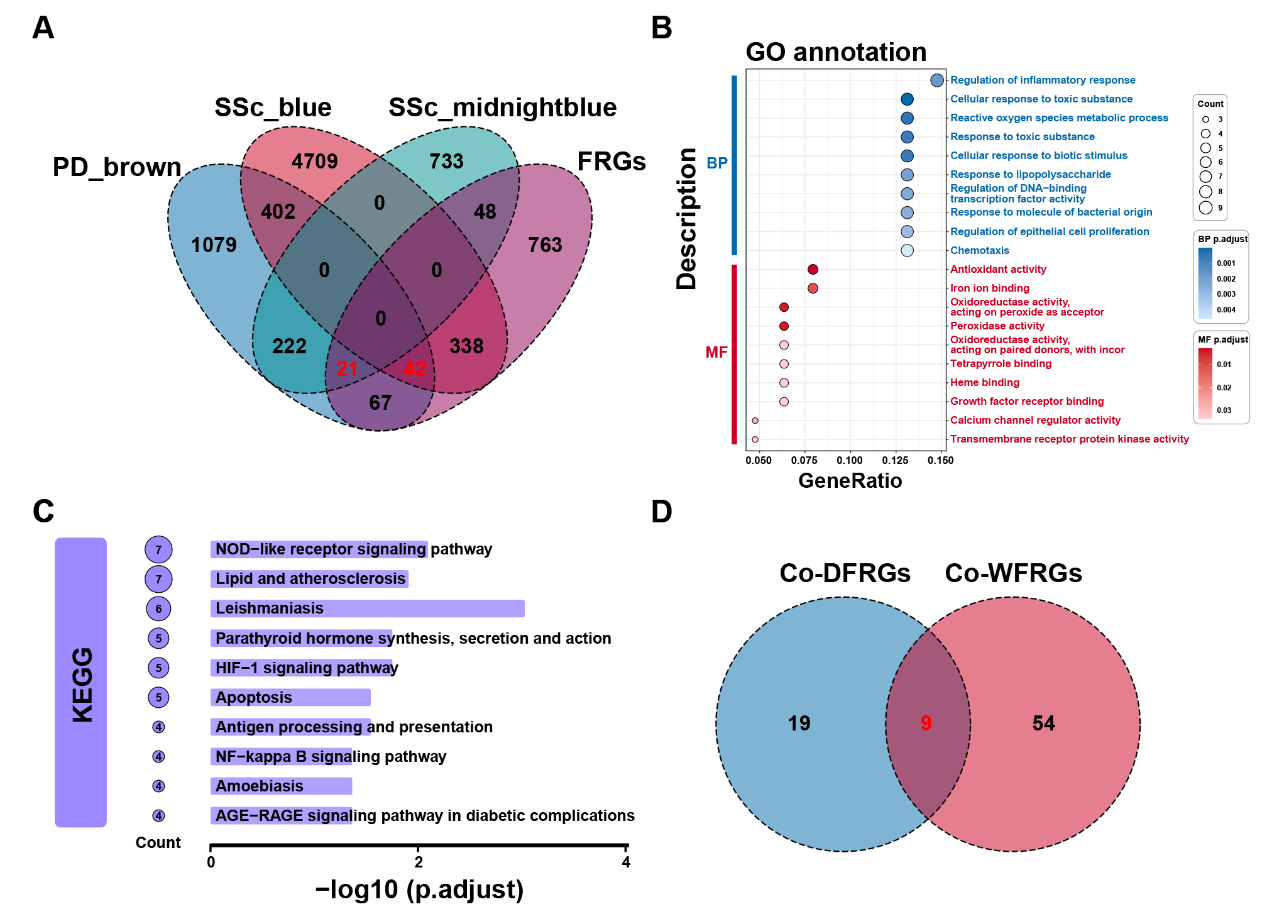


**Functional enrichment of analysis Co-FRMGs. (A)** Venn diagram showing the Co-WFRGs derived from the intersection of FRGs and the key module genes associated with PD and SSc. **(B)** Bubble plots of GO enrichment analysis results for BP and MF. **(C)** Bar plots of KEGG pathway enrichment analysis. **(D)** Venn diagram of overlapping genes in Co-FRDEGs and Co-FRMGs.
